# Multi-omic characterization of the maize GPI synthesis mutant *gwt1* with defects in kernel development

**DOI:** 10.1186/s12870-023-04188-w

**Published:** 2023-04-10

**Authors:** Runmiao Tian, Jianjun Jiang, Shirong Bo, Hui Zhang, Xuehai Zhang, Sarah Jane Hearne, Jihua Tang, Dong Ding, Zhiyuan Fu

**Affiliations:** 1 Key Laboratory of Wheat and Maize Crops Science, Collaborative Innovation Center of Henan Grain Crops, College of Agronomy, Henan Agricultural University, Zhengzhou, 450046 China; 2 CIMMYT, KM 45 Carretera Mexico-Veracruz, El Batan, Texcoco, Edo. De Mexico 56237 Mexico; 3 The Shennong Laboratory, Zhengzhou, 450002 China

**Keywords:** Maize endosperm development, Transcriptomics, Proteomics, Membrane proteomics, GPI anchored protein

## Abstract

**Background:**

Glycosylphosphatidylinositol (GPI) and GPI-anchored proteins (GAPs) are important for cell wall formation and reproductive development in *Arabidopsis*. However, monocot counterparts that function in kernel endosperm development have yet to be discovered. Here, we performed a multi-omic analysis to explore the function of GPI related genes on kernel development in maize.

**Results:**

In maize, 48 counterparts of human GPI synthesis and lipid remodeling genes were identified, in which null mutation of the glucosaminyl-phosphatidylinositol O-acyltransferase1 gene, *ZmGWT1*, caused a kernel mutant (named *gwt1*) with defects in the basal endosperm transport layer (BETL). We performed plasma membrane (PM) proteomics to characterize the potential GAPs involved in kernel development. In total, 4,981 proteins were successfully identified in 10-DAP *gwt1* kernels of mutant and wild-type (WT), including 1,638 membrane-anchored proteins with different posttranslational modifications. Forty-seven of the 256 predicted GAPs were differentially accumulated between *gwt1* and WT. Two predicted BETL-specific GAPs (*Zm00001d018837* and *Zm00001d049834*), which kept similar abundance at general proteome but with significantly decreased abundance at membrane proteome in *gwt1* were highlighted.

**Conclusions:**

Our results show the importance of GPI and GAPs for endosperm development and provide candidate genes for further investigation of the regulatory network in which *ZmGWT1* participates.

**Supplementary Information:**

The online version contains supplementary material available at 10.1186/s12870-023-04188-w.

## Background

Glycosylphosphatidylinositol (GPI) modification is a post-translational modification (PTM) of many membrane proteins that are anchored to the outer cell membrane in eukaryotic organisms [1]. GPI is biosynthesized and preassembled in the endoplasmic reticulum (ER) *via* a multistep pathway. More than 30 genes involved in GPI biosynthesis have been identified and characterized in mammals and yeast [2, 3]. When a GPI biosynthesis gene was mutated, GPI precursor levels were reduced or GPI-anchored proteins (GAPs) were inefficiently transported, which in turn caused cell wall defects in yeast [4]. In humans, 21 genes cover almost all steps of core GPI biosynthesis, GPI maturation, and GPI-protein attachment [5]. Their mutations cause inherited GPI deficiency (IGD), whose main clinical features include global developmental delay, intellectual disability, seizures, hypotonia, and facial dysmorphisms [6, 7]; complete or severe GPI deficiency causes embryonic lethality [8]. In plants, however, very few GPI biosynthesis genes have been reported. *AtSETH1* and *AtSETH2*, homologs of mammalian *PIG-C* and *PIG-A*, are two components of GPI-N-acetylglucosaminyltransferase (GPI-GnT) that transfer N-acetyl-glucosamine to phosphatidylinositol (PI) in the first step of GPI synthesis. Mutations of *AtSETH1* and *AtSETH2* specifically block male gametophyte transmission and pollen function [9]. *AtGPI8* and *AtPIG-S* are two subunits of the GPI transamidase complex (GPI-T) that catalyzes attachment of pre-assembled GPI anchors to GAPs through a transamidation reaction [10, 11]. Loss of function of *AtGPI8* (*gpi8-2*) almost completely abolished male gametophyte function, and null mutations of *AtPIG-S* (*pigs-1*) severely impaired pollen tube emergence and pistil growth. Both these two mutants showed disrupted synergid localization of LORELEI (LRE), a classical GAP [10, 12]. Temperature-dependent intron retention of rice *GPI8* significantly reduced cellulose and lignin content, resulting in a drooping and fragile shoot [13]. *AtPNT1*, the counterpart of mammalian *PIG-M*, encodes a mannosyltransferase that catalyzes the transfer of mannose residues to GlcN-(acyl)PI during GPI biosynthesis. A mutant of *AtPNT1* showed reduced accumulation of AtSKU5, which is an extracellular glycosylphosphatidylinositol–anchored glycoprotein, causing changes in root directional growth and cell wall composition and producing a swollen embryo [14]. Thus, the key genes of GPI biosynthesis are important for plant reproductive development and cell wall composition.

GAPs have similar core backbone structures but diverse functions, acting as hydrolytic enzymes, adhesion molecules, receptors, protease inhibitors, and complement regulatory proteins in humans and participating in various biological processes [15, 81]. In land plants, arabinogalactan proteins (AGPs), a type of extracellular glycoprotein, are a major class of proteins modified by GPI that are involved in the formation of cell wall structural components [16]. In higher plants, about half of the AGP family members contain a GPI lipid anchor [17] and participate in the development and differentiation of stems [18, 19] and roots [20], as well as sexual reproduction [21], embryogenesis [22, 23], fruit ripening [24], and abiotic and biotic stress responses [25, 26]. Fasciclin-like AGPs (FLAs) interact with a range of proteins and have structural and signaling functions in the plant extracellular matrix. Loss of function of FLA16 decreases stem length and alters the biomechanical properties of the mature stem, probably as a result of reduced cellulose levels [19]. The COBRA (a member of the conditional root expansion) family proteins are another class of GAPs that orient microfibrils and deposit cellulose at the cell wall, affecting plant height, leaf and stem brittleness, and development of root hairs, seed coats, pollen, and cotton fiber cells [27–32]. The maize *roothairless3* gene, which influences root hair elongation by an unknown regulatory mechanism [27], and *Bk2L3*, which regulates cellulose deposition, sieve element cell wall ultrastructure, and carbohydrate partitioning, both encode COBRA proteins. Mutations of *Bk2L3* caused leaf chlorosis and hyperaccumulation of starch and soluble sugars in leaves [33]. Several GPI-anchored lipid transfer proteins (LTPGs) with lipid-binding activity, such as rice OsC6 and EPAD1 and wheat TaMs1, are required for maintenance of pollen primexine integrity [34–37]. *Arabidopsis LORELEI* (*AtLRE*) is a maternally expressed imprinted gene that encodes a type of GAP; it mediates maternal control of pollen tube reception and seed development during the transition from the gametophyte to the sporophyte generation [82, 83, 38]. *Arabidopsis* LORELEI-like GPI-anchored proteins 2/3 (LLG2/3) promote pollen tube growth by interacting with ANX/BUPS receptor-like kinases (RLKs), which have been identified as regulators of peptide signal communication processes and may form a receptor–coreceptor complex for perception of RALF peptide signals [39]. Two GPI-anchored aspartic proteases, A36 and A39, play important roles in pollen and ovule development, and their double mutant showed apoptosis-like programmed cell death in pollen grains, degradation of female gametes, and increased abundance of highly methylesterified homogalacturonans and xyloglucans in the apical pollen tube wall [40]. GAPs are thus critical factors modulated by GPI modification during plant cell wall formation and reproductive development. However, the extent to which specific kinds of GAPs are regulated during specific developmental processes is far from being understood in monocots, especially during kernel endosperm development. Identifying GAPs that are regulated by GPI biosynthesis genes during seed development is a necessary step toward resolving this question.

In yeast, *GWT1* encodes an inositol acyltransferase that transfers fatty acyl chains to the inositol moiety of the GPI precursor GlcN-PI [41]. Yeast *Gwt1p* loss-of-function mutants exhibit slow growth and defective cell wall assembly with reduced GAPs [42] or impaired acyltransferase activity and defective export of the detergent-resistant microdomain-associated membrane proteins Tat2p and Fur4p from the ER [43]. In *Cryptococcus neoformans*, *GWT1* is required for laccase repression and stress resistance [44]. However, relatively little is known about plant GWT1 members. Here, we characterized a maize kernel EMS mutant of *ZmGWT1*. To explore the cytology of *ZmGWT1* mutation and investigate what kind of GAPs are involved in kernel development, we investigated kernel cytological features and performed membrane proteomics of wild-type and *gwt1* mutant endosperms. Integration of transcriptomic, proteomic, and membrane proteomic data indicate the significance of specific subcellular proteomic analysis and outline critical GAPs for BETL development in kernels.

## Results

### Characterization of the ***gwt1*** mutant

**Fig. 1:**
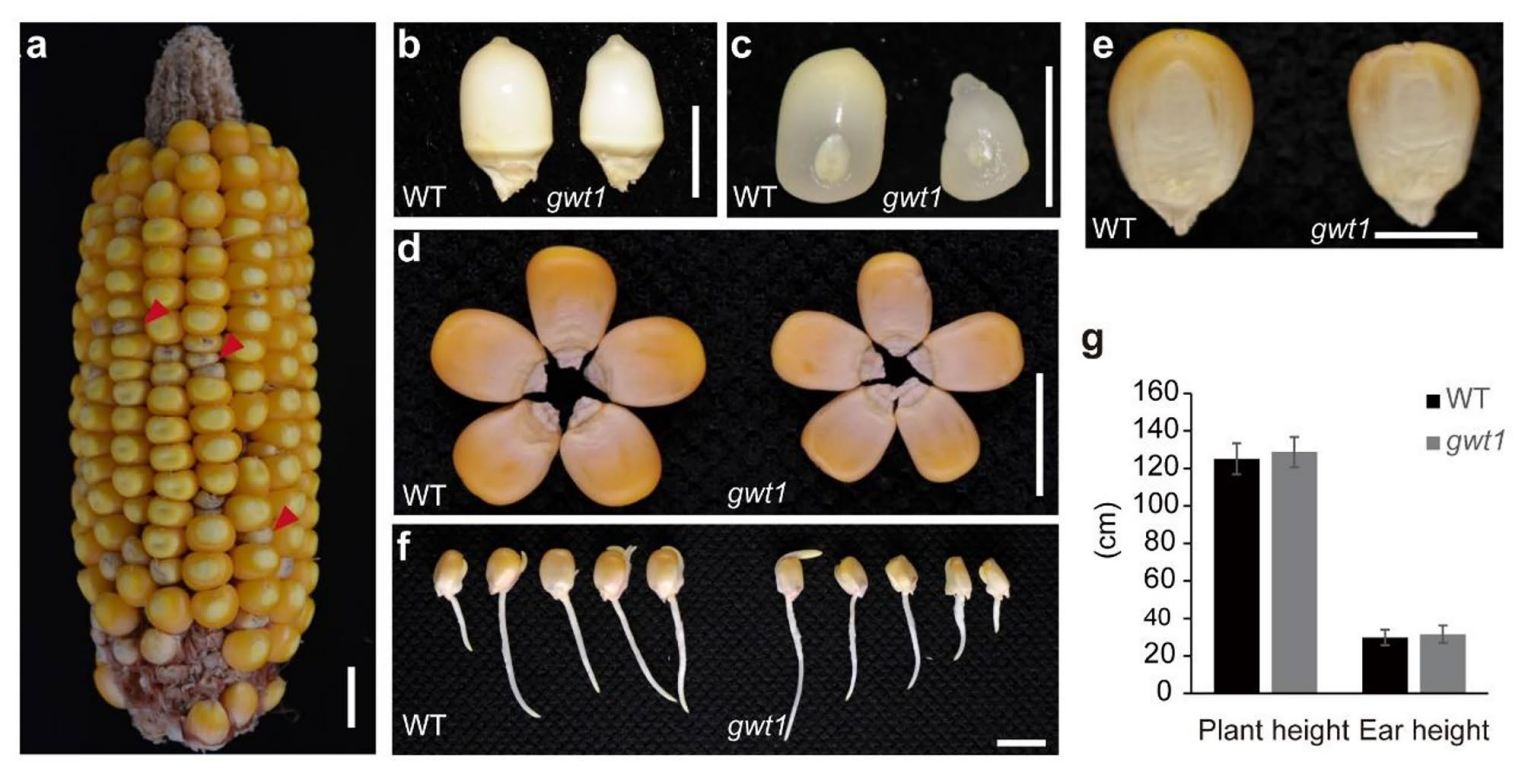
Phenotypic comparison between WT and the gwt1 mutant. **a** Segregating ear of heterozygote (+/-) in B73 background. The red arrowheads indicate *gwt1* kernels. Scale bar, 1 cm. **b-e** Comparison of wild-type (WT) and *gwt1* kernels from the same ear. **b-c** is for kernels of 10 DAP and **d**-**e** is for mature kernels. Scale bar, 1 cm for **d** and 0.5 cm for **b**, **c** and **e**. **f** Germination appearance between WT and *gwt1*. Scale bar, 1 cm. **g** Comparison of plant height and ear height between WT (n = 17) and *gwt1* (n = 11)

**Fig. 2:**
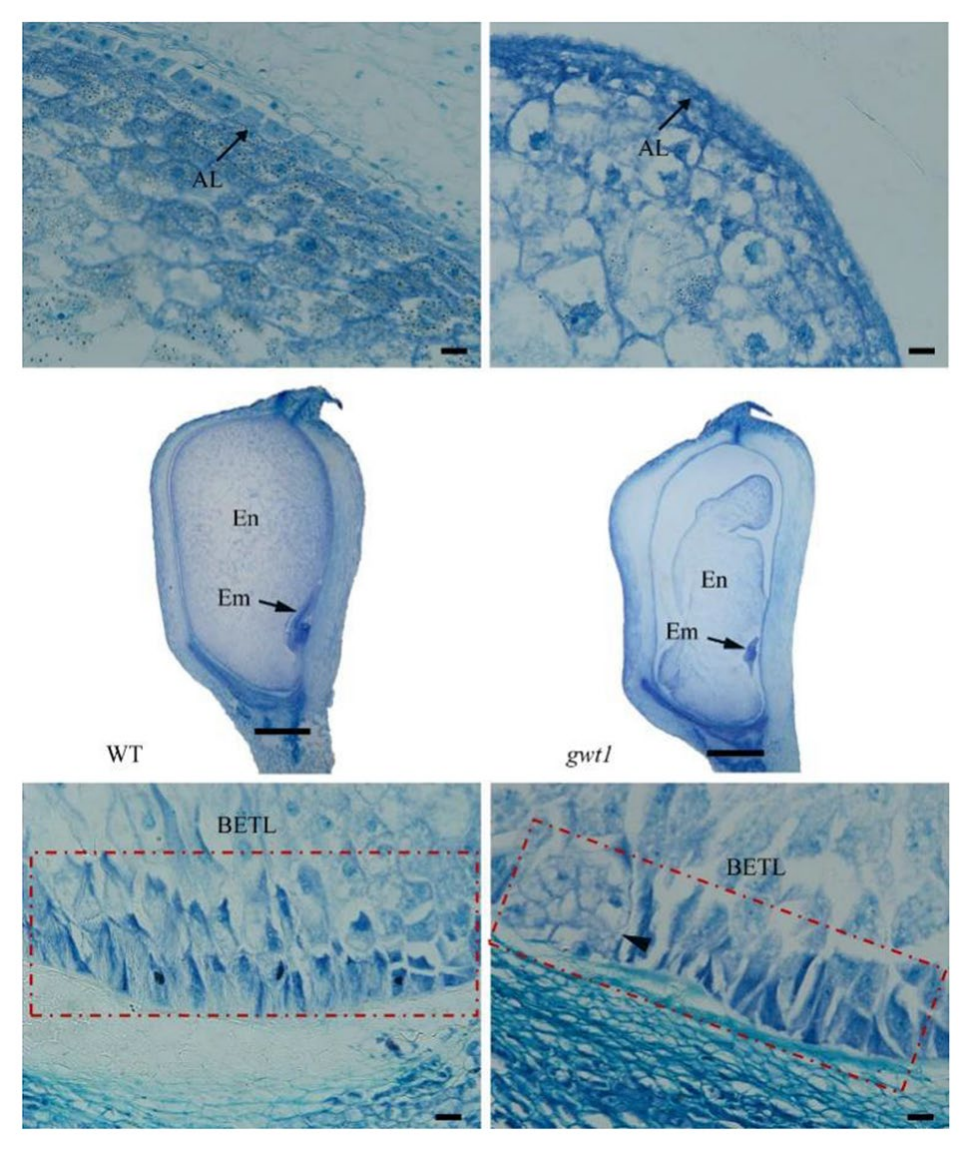
Paraffin sections of WT and *gwt1* kernels at 10 DAP. Scale bars of whole seed, aleurone layer (AL, black arrow) and basal endosperm transfer layer (BETL, red rectangle), 1 mm and 20 μm, respectively. En, endosperm; Em, embryo. The black arrowhead indicated mishappen BETL

To explore the function of key genes in maize GPI biosynthesis pathway, we identified fourty-eight maize counterparts of human reported GPI synthesis and lipid remodeling genes by searching on the online blastp (https://blast.ncbi.nlm.nih.gov/Blast.cgi) using amino acid sequence of each human GPI gene with default parameters or OrthoDB (https://www.orthodb.org) using gene name (Table S1). One glucosaminyl-phosphatidylinositol O-acyltransferase1 gene (*ZmGWT1*) was underlined during maize kernel mutants screening, because its null mutation only caused defective kernel without other abnormal characteristics. To explore the molecular function of *ZmGWT1*, a loss-of-function EMS mutant of *ZmGWT1* (EMS4-16656d) was ordered from the Maize EMS-induced Mutant Database (MEMD) [45]. A C to T mutation on exon 8 was verified in EMS4-16656d (*gwt1*), which leads Glutamine (Q) to a stop codon, resulting in a truncated protein wihtout the GWT1 domain (Fig. S1a, b). To detect whether this single nucleotide mutation is causal for mutated kernel phenotype, we developed a dCAPS marker based on this polymorphism and performed linkage analysis. The gel bands showed that T band appears only in all tested mutant kernels while C band and dual-band appears only in WT kernels, indicating a co-segregation of this mutation with mutant kernel phenotype (Fig. S1c). The *gwt1* had smaller kernels than the WT (Fig. 1a), which was evident at 10 days after pollination (DAP) (Fig. 1b, c). At the mature stage, *gwt1* kernels contained deformed endosperm (Fig. 1d, e). Strikingly, the defective *gwt1* mutant did not differ from the WT in terms of vegetative and reproductive growth (Fig. 1f), resulting in similar plant and ear heights at mature stage (Fig. 1g). Paraffin sections of 10-DAP kernels were observed to investigate the cytological basis for the difference between *gwt1* and WT kernels (Fig. 2). Compared with WT kernels, those of *gwt1* exhibited misshapen basal endosperm transport layer (BETL) cells with fewer cell layers and reduced wall ingrowths (Fig. 2). The aleurone (AL) cells of the WT were regular and darkly stained, whereas the *gwt1* endosperm showed no obvious AL structure similar as the WT (Fig. 2). The BETL is a critical tissue for nutrient transport from maternal tissues to the endosperm [46], and the AL is essential for digestion and remobilization of stored reserves during germination, as well as mineral storage and pathogen defense at maturity [47]. Overall, our results suggested that defects in the BETL and AL were likely to be associated with the small kernel size of *gwt1*.

### Genes related with nutrient reservoir and plasma membrane were underlined in transcriptome

**Fig. 3:**
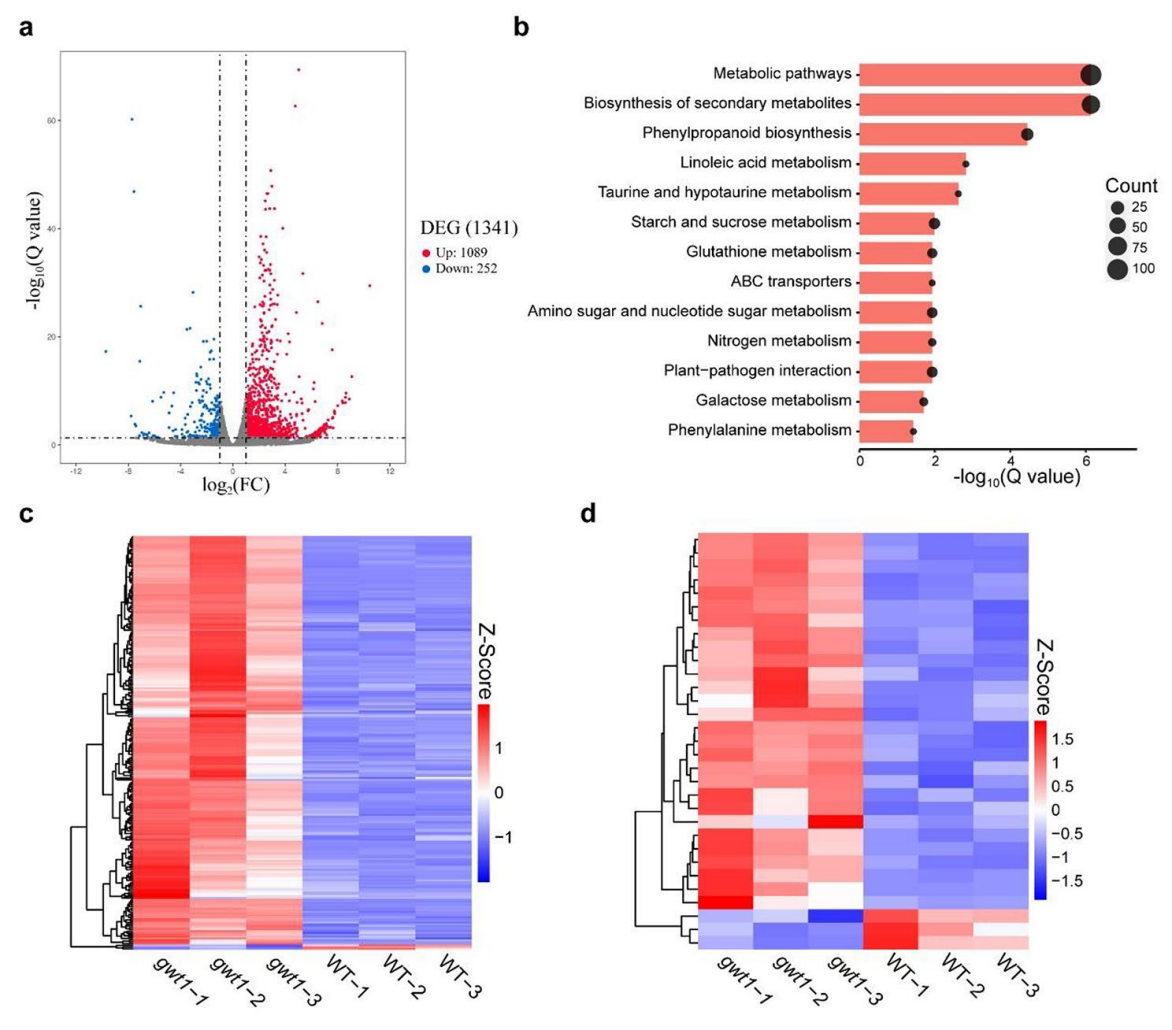
Transcriptomic analysis of 10 DAP endosperm of WT and *gwt1*. **a** Volcano plot for differentially expressed genes (DEGs) between *gwt1* and WT. **b** KEGG analysis of DEGs which are up-regulated in *gwt1* compared with WT. **c** Heatmap for BETL-specific DEGs between *gwt1* and WT. **d** Heatmap for AL-specific DEGs between *gwt1* and WT

To explore genes whose expression changed in response to *ZmGWT1* mutation, we performed RNA sequencing (RNA-Seq) using replicate samples of 10-DAP endosperms from *gwt1* and WT kernels. In total, 25,846 transcripts were identified in at least two of the three replicates. Among them, 1,341 genes were defined as differentially expressed genes (DEGs, fold change > 2, *p* < 0.05) between *gwt1* and WT: 1,089 up-regulated and 252 down-regulated in *gwt1* (Fig. 3a). GO terms were assigned to the up- and down-regulated DEGs by GO enrichment analysis. Among the down-regulated DEGs, lipid biosynthesis (GO:0008610), starch biosynthetic process (GO:0019252), and starch metabolic process (GO:0005982) were the top three biological process GO terms assigned to the largest number of genes (Fig. S2a; Table S2). The top cellular component terms were amyloplast starch grain (GO:0009568), extracellular region (GO:0005576), cell wall (GO:0005618), and external encapsulating structure (GO:0030312) (Fig. S2a; Table S2). Be consistent with the common characteristics in endosperm development defective mutant, the down-regulated genes were enriched in nutrient reservoir activity (GO:0045735) (Fig. S2a; Table S2). However, the down-regulated DEGs couldn’t be significantly enriched in any KEGG pathways (Table S3). For the up-regulated DEGs, stimulus-response GO terms, including response to biotic stimulus (GO:0009607), response to external stimulus (GO:0009605), and response to chemical (GO:0042221) were among the top biological process GO terms assigned to the largest number of genes. The top molecular function GO terms were transmembrane transporter activity (GO:0022857) and hydrolase activity (GO:0016798, GO:0004553) (Fig. S2b; Table S2). Among the most significant cellular component terms, several GO terms were directly related to plasma membrane and cell wall: intrinsic component of plasma membrane (GO:0031226), plasma membrane (GO:0005886), integral component of plasma membrane (GO:0005887), plasma membrane part (GO:0044459), cell wall (GO:0005618) and plant-type cell wall (GO:0009505). KEGG enrichment analysis revealed that the up-regulated DEGs were involved in metabolic pathways such as phenylpropanoid metabolism, starch and sucrose metabolism, and amino sugar and nucleotide sugar metabolism, ABC transporters and plant pathogen interaction (Fig. 3b; Table S3). It is noteworthy that transcription levels of the majority of GPI synthesis genes were almost unaffected except for a 1.86-fold reduction of *ZmGWT1* in *gwt1* (Table S4).

In view of the significant alteration in the BETL and AL between the *gwt1* mutant and the WT (Fig. 2), we therefore focused on DEGs in these two tissues. Based on defined kernel compartment-specific gene information from a previous study [48], 235 DEGs between *gwt1* and the WT were predicted to be expressed specifically in the BETL and 31 in the AL (Table 1 and S4). Furthermore, we observed a high proportion of BETL-specific DEGs (about 44%) compared with other compartment-specific DEGs (each less than 12%) (Table 1). Unexpected, almost all the BETL-specific DEGs, including *MN1*, *MRP1*, *MEG1*, *TCRR-1/2*, and *BETLs* (Table S5), and many AL-specific DEGs were upregulated in *gwt1* (Fig. 3c, d). These results suggest that there may be a feedback regulation of BETL-specific gene expression in response to *ZmGWT1* mutation during endosperm development, with defects in GPI modification of membrane proteins leading to up-regulation of their encoding genes.

**Table 1:** Kernel compartment-specific genes in transcriptome according to previous study

Kernel compartment	Expressed gene number	DEG number	The ratio of DEG (%)
AL	580	31	5.34
BETL	534	235	44.01
CSE	228	8	3.51
CZ	255	9	3.53
EMB	1565	83	5.30
ESR	351	39	11.11
NU	1204	47	3.90
PC	817	50	6.12
PE	1543	79	5.12
PED	1233	88	7.14

### Proteins involved in mitochondrial energy and storage reserves metabolism were prominent

**Fig. 4:**
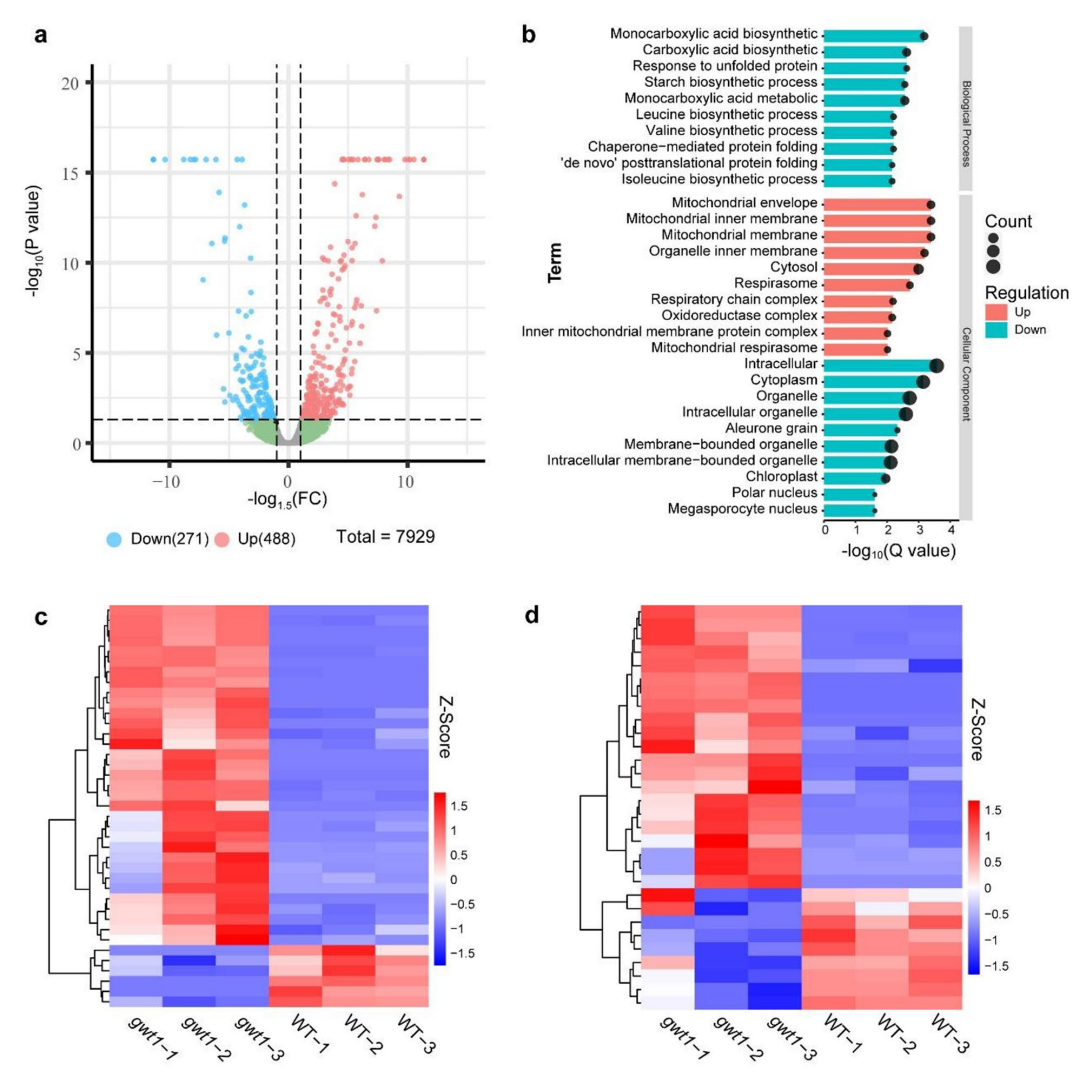
Proteomic analysis of 10 DAP endosperm of WT and *gwt1*. **a** Volcano plot for differentially accumulated proteins between *gwt1* and WT. **b** GO enrichment of differentially accumulated proteins between *gwt1* and WT. **c, d** Heatmap for genes with differences at translation level between *gwt1* and WT. **c** is for BETL-specific expressed genes and **d** is for AL-specific expressed genes

Because the transcriptome does not necessarily reflect gene function at the proteomic level, we characterized the proteome of the RNA-seq samples to document changes at the translational level. In total, 7,929 proteins were successfully identified in *gwt1* and WT in at least two out of three replicates, and only 759 (9.6%) of these were differentially accumulated (1.5-fold cutoff, *p* < 0.05) between *gwt1* and the WT (Fig. 4a). Proteins with lower abundance in *gwt1* (271 proteins) were enriched in biological process GO terms such as carboxylic acid biosynthetic process (GO:0046394), monocarboxylic acid metabolic process (GO:0032787), leucine, isoleucine, and valine biosynthetic processes (GO:0009099, GO:0009098, GO:0009097), starch biosynthetic process (GO:0019252), de novo post-translational protein folding (GO:0051084), and chaperone-mediated protein folding (GO:0051085), and cellular components such as intracellular organelle (GO:0005622) and aleurone grain (GO:0033095) (Fig. 4b). Those with higher abundance in *gwt1* (488 proteins), including three proteins of GPI synthesis or GAP transportation (CLPTM1L, PIGO, and p24beta2; Table S4), were not enriched in any biological process terms but enriched in multiple cellular component terms associated with mitochondria (Fig. 4b). As with the transcriptome results, we again focused on proteins that function in BETL and AL development. We identified 142 BETL-specific proteins and 241 AL-specific proteins based on a previous report [53], 27.46% (39/142) and 12.45% (30/241) of which were differentially accumulated between *gwt1* and WT (Table 2, S6). In accordance with transcriptome, the proportion of DEGs in BETL-specific genes were higher than other compartment-specific genes which imply that BETL was the most affected compartment in *gwt1*. The majority (84.61%) of the BETL-specific differentially accumulated proteins and 70% of the AL-specific differentially accumulated proteins were present at higher levels in *gwt1* (Fig. 4c, d), including Mn1 and BETL2. However, BETL10 and TCRR-2 levels were lower in *gwt1*. These results suggest that *ZmGWT1* had a greater effect on BETL-specific proteins than on other compartment-specific proteins during endosperm development.

**Table 2:** Kernel compartment-specific genes in proteome according to previous study

Kernel compartment	Accumulated protein number	Differentially accumulated protein number	The ratio of differentially accumulated protein (%)
AL	241	30	12.45
BETL	142	39	27.46
CSE	114	1	0.88
CZ	96	12	12.5
EMB	369	31	8.40
ESR	79	14	17.72
NU	349	32	9.17
PC	190	29	15.26
PE	320	36	11.25
PED	299	36	12.04

**Fig. 5:**
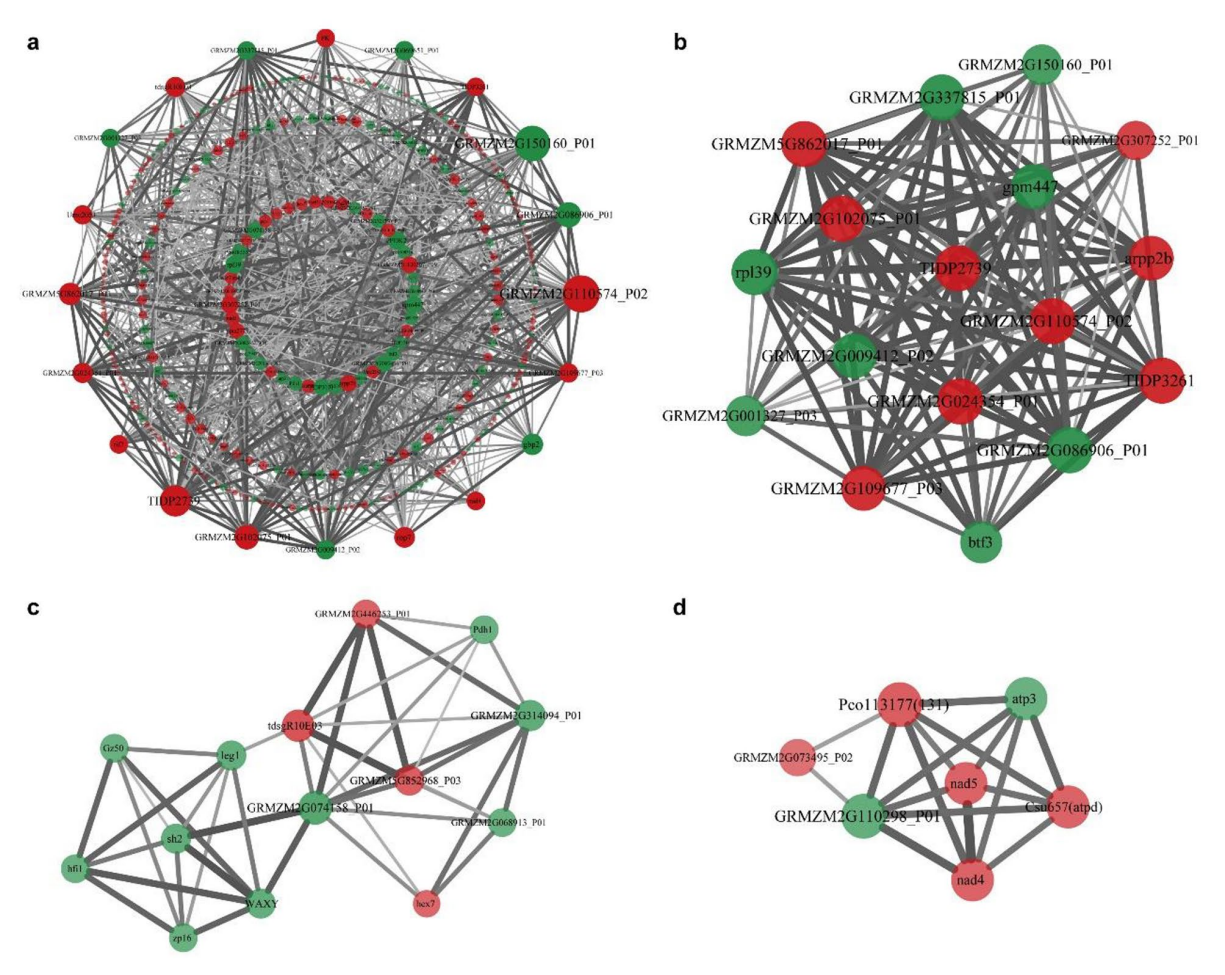
Protein-protein interaction (PPI) network of differentially accumulated proteins identified in the proteome. **a** PPI network constructed using the differentially accumulated proteins. Number of nodes: 345; number of edges: 1101; average node degree: 4.63; avg. local clustering coefficient: 0.321; expected number of edges: 971; PPI enrichment p-value: 2.35E-05. The red nodes stand for proteins with higher abundance in *gwt1*, while the green nodes stand for lower abundant proteins in *gwt1*. **b-d** The significant modules were retrieved from the PPI network using the molecular complex detection (MCODE) method with a score of ≥ 5.0. **b** shows module 1 with an MCODE score of 15.750, **c** shows module 2 with an MCODE score of 6.380, and **d** shows module 2 with an MCODE score of 5.667. The red nodes stand for proteins with higher abundance in *gwt1*, while the green nodes stand for lower abundant proteins in *gwt1*

To test whether the differentially accumulated proteins were involved in a common module, we performed a protein–protein interaction (PPI) network analysis. Using a medium confidence cut-off (confidence score > 0.4), 345 differentially accumulated proteins were identified as network nodes (Fig. 5a). The number of connections in the PPI network constructed from differentially accumulated proteins was significantly higher than that of a network constructed from randomly selected proteins (P = 2.35e − 05), indicating that the differentially accumulated proteins were at least partially biologically related. Three significant modules with scores ≥ 5 (modules 1, 2, and 3) were extracted from this network using MCODE (Fig. 5b, c, d). Proteins in module 1 were enriched in translation (GO:0006412) and related cellular component GO terms such as ribosome (GO:0005840) (Fig. S3). Proteins in module 2 were significantly enriched in terms related to carbohydrate metabolism such as glycolytic process (GO:0006096), carbohydrate metabolic process (GO:0005975), generation of precursor metabolites and energy (GO:0006091), and starch and sucrose metabolism (GO:0019252), most of which were present at lower levels in *gwt1* (Fig. S4). Proteins in module 3 were mainly enriched in energy- and mitochondrion-related terms such as ATP metabolic process (GO:0046034), mitochondrion (GO:0005739), mitochondrial membrane (GO:0031966), and respirasome (GO:0070469), most of which were abundant in *gwt1* (Fig. S5). These PPI results further emphasized the strong influence of *ZmGWT1* on mitochondrial energy metabolism and storage reserves metabolism.

**Fig. 6:**
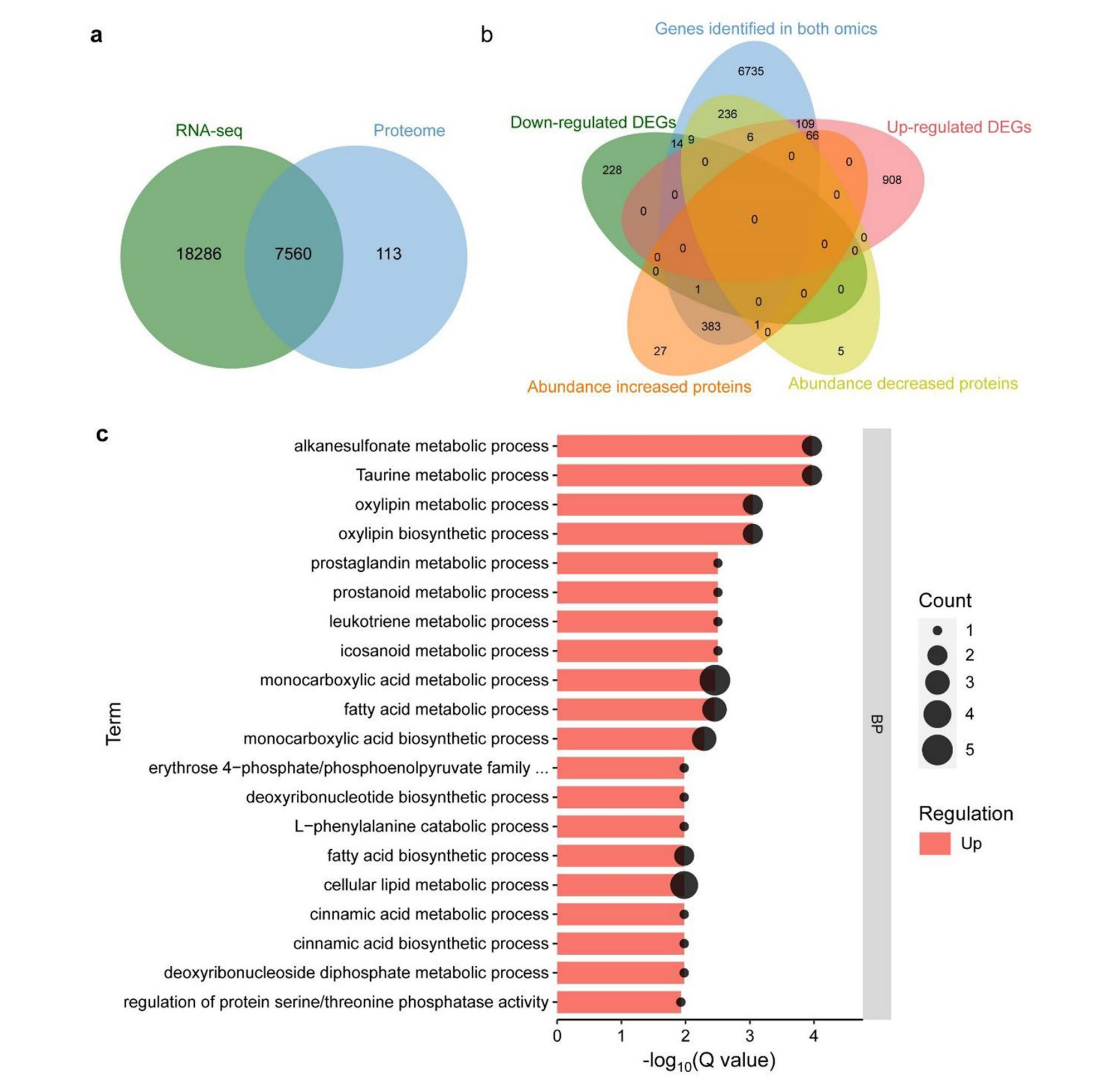
Integrative analysis of transcriptome and proteome. **a, b** Venn diagram for overlapped gene number and DEG number between transcriptome and proteome. **a** is for total genes and **b** is for DEGs. **c** GO enrichment for common changed genes at both transcription and translation levels between *gwt1* and WT

When compared with the transcriptome, 7,560 of the 7,929 protein-coding genes were also identified at the transcriptional levels (Fig. 6a), but only 1% (82/7,929) of them were differentially expressed at both levels (Fig. 6b; Table S7). Sixty-six out of the 82 proteins were increased at both levels in *gwt1*; their annotations indicated that they were involved in secondary metabolite metabolism and synthesis, including monocarboxylic acid biosynthetic process (GO:0072330) and metabolic process (GO:0032787), fatty acid biosynthetic (GO:0006633) and metabolic process (GO:0006631), and cellular lipid metabolic process (GO:0044255) (Fig. 6c). Only nine of the 82 proteins were decreased at both levels in *gwt1*, and seven proteins showed abundance changes opposite to their gene expression changes (Fig. 6b; Table S7). These results further underscored the inconsistence of transcriptome with proteomics and the necessity of exploring molecular mechanism at different omics levels.

### Important GAPs involved in kernel development

**Fig. 7:**
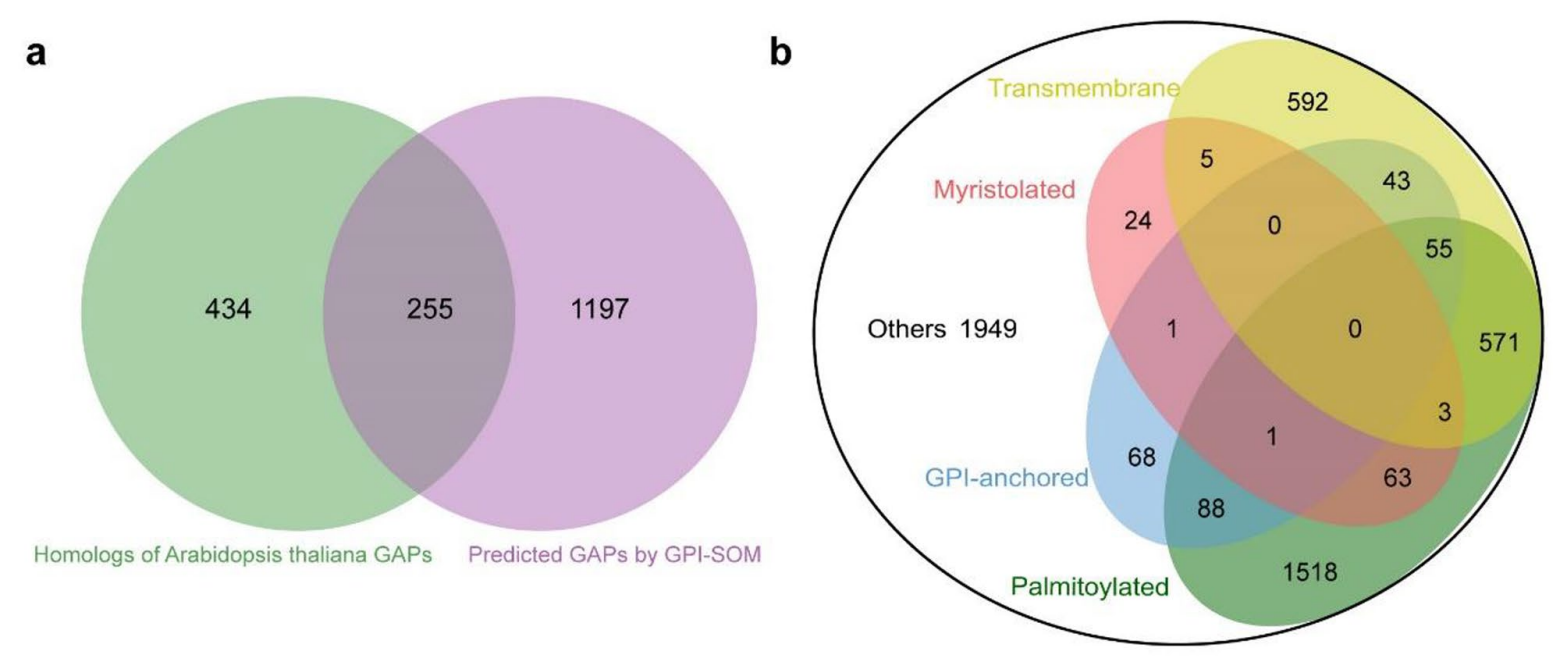
GAPs mining from membrane proteome. **a** Venn diagram for predicted common GAPs using two methods in maize. **b** Classification of membrane proteins according to transmembrane and lipid modification structures prediction

Because glycosylphosphatidylinositol (GPI) modification is an important post-translational modification (PTM) of many membrane proteins, we therefore performed a specific membrane proteome of kernels to identify difference of GPI-anchored proteins (GAPs) between *gwt1* and WT. To define specific GPI modification proteins, we further annotated maize B73 gene models using GPI-SOM prediction, meanwhile we also searched for the homologs of *Arabidopsis thaliana* reported GAPs by OrthoFinder2. 1,452 potential GAPs and 689 maize counterparts of *Arabidopsis* GAPs were retrieved (Table S8), which were derived from 1,886 nonredundant genes (Fig. 7a). 256 predicted GAPs were included in the 4,981 identified proteins in membrane proteome, which along with the proteins with transmembrane domain, palmitoylation, or myristoylation modification composed of 3,032 predicted membrane proteins (Fig. 7b; Table S9). 18% (47/256) of the potential GAPs were differentially accumulated between *gwt1* and WT, which were enriched in GO terms of transport and metabolic process (Table S10). Among them, twenty-four GAPs with decreased abundance in *gwt1* (Table S10), such as the classical GAPs, early nodulin-like proteins, plasmodesmata callose-binding protein, and lipid transfer protein involved in reproductive growth and seed development in *Arabidopsis* and rice.

To narrow down the searching targets for *ZmGWT1* regulation model, we classified the 256 potential GAPs into compartment-specific ones according to their expression pattern in kernels [48], AL (9), BETL (9), CSE (4), CZ (10), EMB (11), ESR (3), NU (18), PC (8), PE (15), PED (7) ( Table S11). GAPs coding genes that are BETL- and/or AL-specific were outlined, because the observed significant defects in BETL and AL of *gwt1* mutant. 44.4% and 33.3% of them were differentially accumulated between *gwt1* and WT, including two BETL-specific and two AL-specific GAPs with increased abundance and two BETL-specific and one AL-specific GAPs with decreased abundance in *gwt1*. In addition, accumulated GAPs difference couldn’t project its change in total protein level because GPI is a kind of PTM, and null mutation of *ZmGWT1* might reduce GAPs specifically on membrane, we therefore focus on proteins that showed decreased abundance in *gwt1* membrane proteome but with unchanged abundance levels with the WT in total proteome (Table 3). Consequently, two BETL-specific genes, *Zm00001d018837* and *Zm00001d049834*, encoded proteins were revealed with 43- and 78-fold decrease in membrane protein abundance in *gwt1* (Table S10), providing crucial candidate genes influencing kernel development in *ZmGWT1* regulation module.

**Table 3:** Decreased abundance GAPs in *gwt1* specifically at PM

Accession	GeneID	Compartment-specific pattern	Description
B4F845	Zm00001d045336		Uncharacterized protein
A0A1D6I6P5	Zm00001d020867	ESR	Subtilisin-like protease SBT5.3
B6UI23	Zm00001d016741		Pectinesterase inhibitor domain containing protein
B4FEN4	Zm00001d002148	-	Syntaxin-51
B6TQP9	Zm00001d031636	NU	Lipid transfer protein
A0A096QAE9	Zm00001d018837	BETL	BURP domain protein RD22
A0A1D6L558	Zm00001d034062		Plasmodesmata callose-binding protein 2
C0HIK6	Zm00001d037756	PED	Zinc transporter 4
A0A1D6E4P3	Zm00001d002835	CSE	Putative LRR receptor-like serine/threonine-protein kinase
B6TQP7	Zm00001d049834	BETL	Lipid transfer protein
C0P7H1	Zm00001d036482		Core-2/I-branching beta-16-N-acetylglucosaminyltransferase family protein

## Discussion

The BETL is a unique endosperm cell layer with cell wall ingrowths, which increase the plasma membrane surface area of the BETL cells and critical for effective nutrient transport from the maternal placenta to the developing endosperm [49–51]. Many reported kernel mutants exhibit decreased BETL cells and cell wall ingrowth, such as *mn1* [52, 53], *ZmSWEET4c* [54] and *Smk10* [51]. AL is another important endosperm cell layer, and mutants with disrupted AL cell differentiation, such as *dek1*, *Nkd1*, and *Nkd2*, generally exhibit abnormal endosperm development [55, 56]. Here, we characterized a *gwt1* mutant with deformed endosperm that contained misshapen BETL cells and exhibited a defective AL structure. Consistent with these cytological observations, we found that the BETL-specific genes were the most affected compartment-specific genes at transcriptome (44.01%), proteome (27.46%), and PM proteome (33.33%) (Tables 1 and 2 and S12). Although AL was also a defective part at cytologic levels, the AL-specific genes were relatively little affected at different omic levels by *ZmGWT1* mutation compared with BETL-specific genes, 5.34% at transcriptome, 12.45% at proteome and 13.86% at membrane proteome (Tables 1 and 2 and S12). The results indicate that *ZmGWT1* mainly influence the function of GAPs in the BETL to modulate kernel development.

PM proteins are generally underrepresented in proteomic analyses because of their relatively low abundance compared with soluble proteins or other endomembrane proteins [57, 58]. Subcellular proteome analysis is one of the most effective ways to reduce the complexity of the total proteome. For example, we identified a further 1,526 proteins in the membrane proteome that were missing from the total proteome (Fig. S6), including 254 differentially accumulated predicted PM proteins (Table S12). Strikingly, differentially accumulated PM proteins were enriched in terms transporter activity (GO:0005215, 18/254), transport (GO:0006810, 16/254), and transmembrane transport (GO:0055085, 13/254), in consistent with the cytosolic characterization in BETL of *gwt1* (Fig. S7). Therefore, it is necessary to perform subcellular proteome for special post-translational modification. In recent, only few previous studies on maize plasma membrane proteome were reported. Hopff identified 298 plasma membrane proteins from 18-day-old maize roots grown under low and high iron conditions using mass spectrometry [59]. Using plasma membrane-enriched proteomics, Zhang identified 686 plasma membrane proteins that corresponded to 649 UniProtKB IDs [60], and Voothuluru identified 3,462 plasma membrane proteins that corresponded to 3,193 UniProtKB IDs [61] from the primary root growth zone of maize (Table S14). One hundred ninety-five membrane proteins identified in the present study were also identified in the latter two reports (Fig. S8). The tissue specificity of gene expression may be a major reason for the differences in membrane proteins among these three studies, but differences in growth conditions and plasma membrane protein extraction methods may also have had an effect.

*gwt1* is a null mutant of *ZmGWT1* that exhibited defective BETL and great changes in membrane proteins. Several GAP-coding gene with decreased abundance, including early nodulin-like proteins, plasmodesmata callose-binding proteins, and lipid transfer proteins were emphasized, especially two predicted BETL-specific GAPs, BURP domain protein RD22 (*Zm00001d018837*) and lipid transfer protein (*Zm00001d049834*) [48]. Nonspecific lipid transfer proteins (nsLTPs) are small, basic proteins, characterized by eight conserved Cys residues [62]. nsLTPs are involved in a wide range of biological functions, such as the development of pollen [36], seed [63] and seed coats [64]. *Arabidopsis* type I LTPs (nsLTPs with GPI site, AtLTPG1, AtLTPG2 and AtLTPG6) are mainly expressed in flower and seed, which would primarily be involved with synthesis or deposition of cuticular lipids [65, 66]. A mutation in *AtLTPG1* resulted in reduced alkane accumulation at the plant surface, suggesting its involvement in lipid export [67, 68]. Knockout mutants of *AtLTPG2* and *AtLTPG6* showed early aborted seeds and infertile ovules compared with the wild type, suggesting that these gene also participate in seed development [64]. In other hand, LTPs were thought to influence membrane biogenesis [69]. *Zm00001d049834* is one of the homologs of *AtLTPG1* and *OsLTPG22*, and the protein abundance of its paralogue (*Zm00001d031636*) was also significantly decreased (19-fold change) at PM proteome in *gwt1* (Table S10). Thus, we speculate that *Zm00001d049834* may participate in wall ingrowth formation, and its reduced abundance in *gwt1* at PM might be associated with BETL defects. The BURP domain-containing protein (BURP protein) is a plant-specific protein that contains a conserved BURP domain at the C-terminus. RD22-like gene is found to associated with abiotic stresses and plant development [70, 71]. GhRDL1 is localized in the cell wall and interacts with GhEXPA1 to loosen the cell wall and increase seed size in cotton, and epotic expression of *GhRDL1* in *Arabidopsis* also produces a substantial increase in seed size [72]. However, the function of its maize counterpart is still unknown. Additionally, no experimental evidence show that these two proteins were GAPs in monocots and their function on BETL development are still unrevealed. Together, our results provide potential GAPs regulated by *ZmGWT1*, which might influence kernel development through their potential function on BETL wall ingrowth formation.

## Materials and methods

### Plant materials

The *gwt1* EMS mutant (EMS4-16656d) in the B73 background was ordered from the Maize EMS-induced Mutant Database (MEMD) (http://www.elabcaas.cn/memd/) [45]. There is a C to T mutation (SNP) in this mutant. To confirm the mutation site in the offspring of its heterozygotes (+/*-*), normal kernels and mutant kernels from the same segregating ear was sequenced with their separate DNA pool. A dCAPS marker based on this SNP was developed to test the linkage of marker and phenotype, in which only the wild type (WT) PCR product can be cleaved by enzyme *Sac* I. Heterozygous +/*-* individuals were planted and manually pollinated for three generations to obtain homozygous +/+ (wild type, WT) and -/- (*gwt1*) kernels for omics analysis. Endosperm samples without pericarp and embryo were collected from kernels at 10 days after pollination (DAP). For all measurements, three biological replicates were obtained from each genotype, and each replicate consisted of five ears. To exclude environmental effects, only kernels from the middle section of each ear were excised. All materials were grown at the research farm of Henan Agricultural University, Zhengzhou, China (113°420 E, 34°480 N).

### Cytological sections

Paraffin sections of 10-DAP kernels were prepared according to a previously described protocol [73]. The samples were treated with xylene, embedded in paraffin wax *via* infiltration, and cut into 10 μm-thick sections with a Thermo Scientific HM 325 rotary microtome. The sections were stained with toluidine blue (Sinopharm Chemical Reagent Co., Ltd) and examined under a ZEISS Stemi 508 Stereo Microscope (Germany) and a ZEISS Axio Scope A1 Microscope (Germany).

### RNA-seq library construction and transcriptome sequencing

Total RNA was extracted from three replicate 10-DAP endosperm samples of *gwt1* and WT using the RNAprep Pure Plant Kit (Tiangen). RNA-seq libraries were constructed with the VAHTSTM Stranded mRNA-seq Library Prep Kit for Illumina (Vazyme). These libraries were sequenced on an Illumina NovaSeq platform, using 150-bp paired-end reads, and 6 G sequencing depth. RNA purity and concentration were assessed using a NanoDrop 2000 spectrophotometer, and RNA integrity and quantity were examined with an Agilent 2100/4200 bioanalyzer system. Paired-end clean reads were aligned to the B73 reference genome using HISAT2. The number of clean reads that mapped to each gene was counted using FeatureCounts[74]. Differentially expressed genes (DEGs) between the WT and the *gwt1* mutant were identified using the edgeR Bioconductor package based on the criteria of (1) greater than two-fold expression change and (2) corrected p value < 0.05 [75]. GO and KEGG enrichment analyses were performed using the topGO (http://www.bioconductor.org/packages/release/bioc/html/topGO.html) and KOBAS packages [76], respectively.

### Proteome analysis

Each sample was ground in liquid nitrogen and transferred to a 2-mL centrifuge tube. After sonication in lysis buffer (50 mM Tris–HCl, 10 mM DTT, 1 M sucrose, 1% Triton X-100, 1% protease inhibitor cocktail, and 1 mM EDTA) for three minutes on ice, an equal volume of Tris-saturated phenol (pH 8.0) was added, and the mixture was vortexed for 5 min. The upper phenol phase was transferred to a new centrifuge tube after centrifugation at 5,000 g for 10 min at 4 °C. Proteins were precipitated using four volumes of 100 mM ammonium acetate in methanol and incubated overnight at − 20 °C. The supernatant was discarded after centrifugation for 10 min at 4 °C, and the remaining precipitate was washed with ice-cold methanol (once) and ice-cold acetone (three times). The pellet was redissolved in 8 M urea, and the protein concentration was measured using a Bradford kit (Thermo Scientific) according to the manufacturer’s instructions.

For digestion, the purified protein solution was diluted with 5 mM DTT for 30 min at 56 °C and alkylated with 11 mM iodoacetamide (IAM) for 15 min at room temperature in the dark. The solution was then transferred to an ultrafiltration tube and centrifuged at 12,000 g at room temperature for 20 min. Urea was then replaced with ammonium bicarbonate (ABC), and the mixture was centrifuged three times. Trypsin was added at a ratio of 1:50 (protease: protein, m/m) and incubated at 37 °C overnight. The peptides were recovered twice with ABC by centrifugation at 12,000 g for 10 min at room temperature.

The tryptic peptides were dissolved in buffer A (2% ACN, 0.1% FA) and centrifuged at 20,000 g for 30 min. The supernatant was transferred to a sample tube and loaded onto a silica capillary column (75 μm ID, 360 μm OD) on an EASY-nLC 1200 nanoLC system (Thermo Scientific), then pulled to a 10-µm, 18-cm length column packed with 1.9-µm beads (Reprosil-Pur C18-AQ, Dr. Marisch GmBH). A 120-min gradient was run at 300 nL min^− 1^: 2–8% buffer B (0.1% formic acid in 98% acetonitrile) over 2 min, 8–30% over 110 min, 30–80% over 4 min, and holding at 80% for the last 4 min.

The peptides were exposed to a nano-spray ionization source, followed by tandem mass spectrometry (MS/MS) using an Orbitrap Exploris 480 instrument with a FAIMS Pro interface (Thermo Scientific) coupled online to an ultra performance liquid chromatograph (Thermo Scientific). The specific parameters were: −45 V and − 65 V for FAIMS, 1.8 kV for electrospray voltage, 350 to 1500 for full m/z scan, 60,000 at m/z 200 for full MS resolution, 300% with an IT of 50 ms for MS AGC target, 75% with a resolution of 15,000 and 22 ms injection time with 1 s Top Speed for AGC target value of fragment spectra, 5E4 for intensity threshold, 1.6 m/z for isolation width, and 30% for normalized collision energy.

The MS/MS data were processed using Proteome Discoverer 2.4 software (Thermo Scientific) using the SEQUEST HT search engine. Tandem mass spectra were searched against the *Zea mays* UniProt database using the following criteria: (1) a maximum of two trypsin/P cleavage; (2) a fragment ion mass tolerance of ± 0.02 Da; (3) carbamidomethylation of cysteine residues as a fixed modification and oxidation of methionine residues and N-term acetylation as dynamic modifications. FDR was adjusted to < 1%.

### Membrane proteome analysis

Membrane proteins were extracted from 10-DAP endosperms of the *gwt1* mutant and the WT using the Minute Plasma Membrane Protein Isolation Kit for Plants (Invent Biotechnologies) according to the manufacturer’s instructions. Two hundred millimolar dithiothreitol (DTT) solution was added to 100 µg protein to give a final DTT concentration of 10 mM, and the mixture was incubated at 30 °C for 1 h. Forty millimolar IAM (Amresco, M216-30G) was added, followed by incubation at room temperature for 45 min. The sample was then diluted four-fold using 25 mM ABC buffer. The retrieved proteins were digested with trypsin (trypsin: protein = 1:50) at 37 °C overnight, and digestion was terminated with 50 µL 0.1% FA. A C18 column was sequentially washed with 100 µL 100% ACN and 100 µL 0.1% FA with a 3-min centrifugation at 1,200 rpm for each wash. The column was placed in a new EP tube before sample addition (≤ 30 µg) and centrifuged at 1,200 rpm for 3 min. The column was then washed twice with 100 µL 0.1% FA with 3-min centrifugation at 1,200 rpm before washing once with 100 µL H_2_O (pH 10). Finally, the column was eluted with 70% ACN into a new EP tube, and proteins were lyophilized and stored at − 80 °C until loading.

The lyophilized fraction was dissolved in 10 µL mobile phase A (0.1% formic acid in water) and centrifuged at 14,000 g and 4 °C for 20 min. One microliter of supernatant was loaded. The LC gradient was as follows: 6–12% solvent B (0.1% formic acid in ACN) over 8 min, 12–30% over 50 min, 30–40% over 12 min, 40–95% over 1 min, and holding at 95% for 7 min. Label-free mass spectrometry was performed with a Thermo Orbitrap Fusion mass spectrometer. The scan events were set as a full MS scan of 300–1400 m/z at a mass resolution of 120,000 (200 m/z), followed by a CID MS/MS scan repeated on the 20 most intense ions selected from the previous full MS scan with an isolation window. The normalized collision energy was set to 35%, with an activation time of 50 ms. The second stage used linear ion trap fast mode for data acquisition, an automatic gain control (AGC) of 5,000, a maximum injection time of 35 ms, and a dynamic exclusion time of 20 s. Spectra were searched against the UniProt *Z. mays* database (2020.8.13 Download), and the MS/MS data were processed using MaxQuant 1.5.2.8.

Identification criteria were as follows: (1) a precursor ion mass tolerance of ± 15 ppm; (2) a fragment ion mass tolerance of ± 0.02 Da; (3) a max missed cleavages of 2; and (4) carbamidomethylation (57.021 Da) of cysteine residues as a static modification and oxidation of methionine residues (+ 15.995 Da) as a dynamic modification. Proteins that showed ≥ 1.5-fold changes in accumulation at *p* ≤ 0.05 were used for further analysis.

### Prediction of GPI-anchored proteins

To annotate maize GAPs, the longest peptide sequences of all maize genes were downloaded from NCBI and used as queries for GAP prediction with GPI-SOM (http://genomics.unibe.ch/cgi-bin/gpi.cgi). At the same time, OrthoFinder2 was also used to identify maize counterparts of Arabidopsis GAPs with default parameters [77, 78]. The default sequence search method in OrthoFinder2 is DIAMOND. To classify membrane proteins, TMHMM 2.0 (https://services.healthtech.dtu.dk/service.php?DeepTMHMM) was used to analyze their different structural domains, and CSS-Palm (http://csspalm.biocuckoo.org) and NMT-The MYR Predictor (https://mendel.imp.ac.at/myristate/) were used to predict palmitoylation and myristoylation modifications, respectively. Predicted interaction networks generated with STRING (https://cn.string-db.org/) were visualized in Cytoscape [79], Only interactions with a combined score > 0.4 of all associations obtained in STRING were selected to construct the PPI network. The Molecular Complex Detection (MCODE) plugin in Cytoscape was then utilized to find clusters of the PPI network [80].

### Classification of compartment-specific genes

The classification based on a previously study which provide a high-resolution atlas of gene activity in the compartments of the maize kernel[48]. They identified mRNAs that specifically accumulate in each compartment at 8 DAP by applying a compartment specificity (CS) scoring algorithm to the genes with FPKM ≥ 2 in at least one compartment, those compartments including AL, BETL, embryo-surrounding region (ESR), embryo (EMB), nucellus (NU), placento-chalazal region (PC), pericarp (PE), central starchy endosperm (CSE), the conducting zone (CZ) of starchy endosperm, and the vascular region of the pedicel (PED). And genes with CS score > 0.3 were defined as being expressed in a compartment-specific pattern.

## Electronic supplementary material

Below is the link to the electronic supplementary material.

Supplementary Material 1

Supplementary Material 2

Supplementary Material 3

## Data Availability

The data that support the findings of this study are available upon request from the corresponding author.
