# Supplementary material for: Multi-omic characterization of the maize GPI synthesis mutant gwt1 with defects in kernel development
Source: BMC Plant Biol. 2023 Apr 10;23:191. doi: 10.1186/s12870-023-04188-w (PMC10084604; doi:10.1186/s12870-023-04188-w)
Supplement: Supplementary file 3 — Supplementary Material 3 [file 12870_2023_4188_MOESM3_ESM.pdf]

Supplementary figures

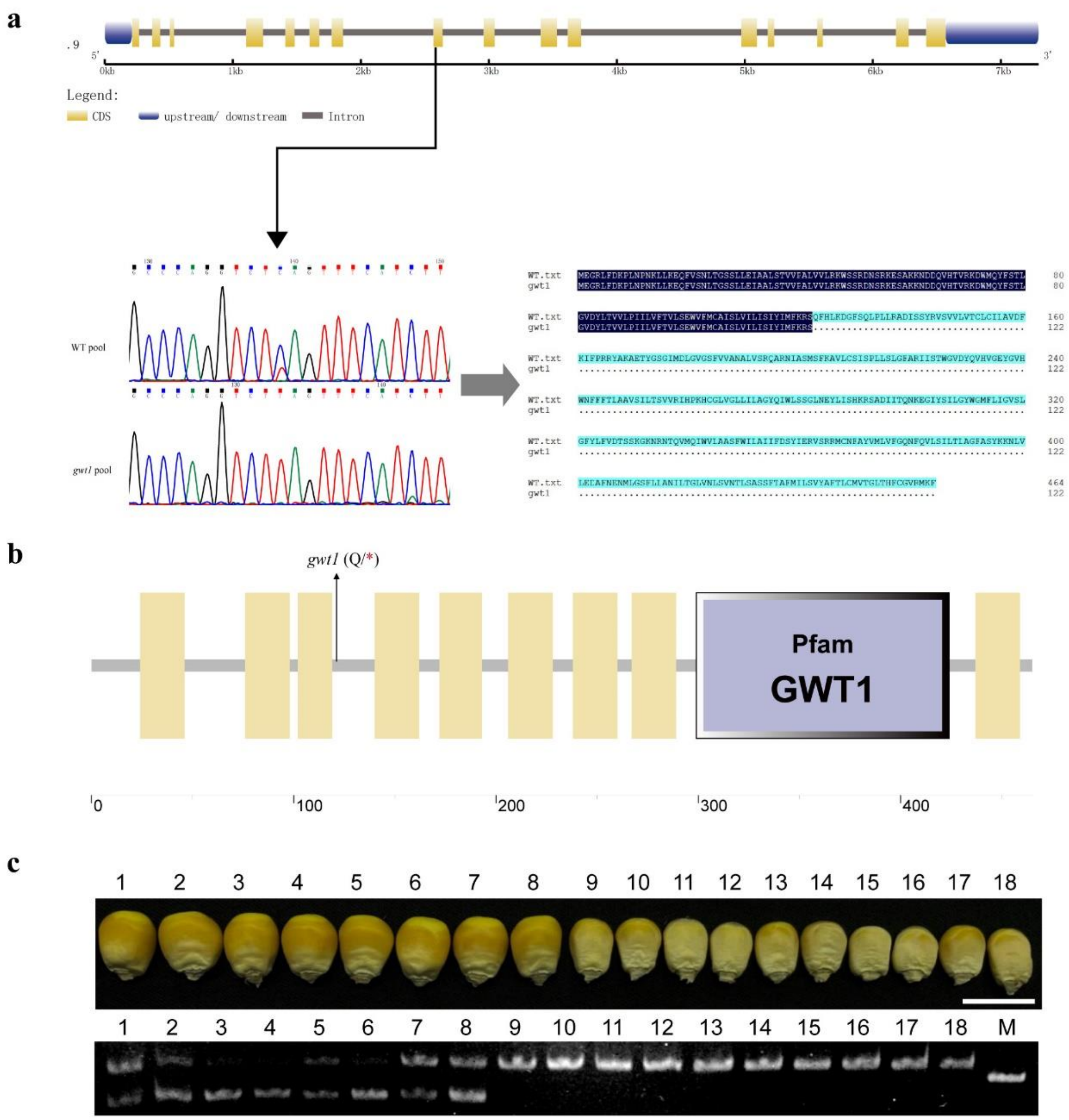

Fig. S1 Mutation site detection and linkage analysis with *gwt1* phenotype. **a** Identification of the mutation site in *gwt1* EMS mutant. The sequences alignment of mutated sites between the normal pool and the *gwt1* pool is on the left. Each pool was DNA mixture extracted from five randomly selected normal kernels and mutant kernels on segregating ear of *gwt1* heterozygote (+/-), respectively. The amino acid alignment of ZmGWT1 between WT and *gwt1* is on the right. **b** Protein structural models of ZmGWT1. The stop codon gain mutation site was marked. The purple rectangle represents GWT1 domain, and the yellow boxes represent transmembrane domain. **c** Linkage analysis of *ZmGWT1* with kernel phenotype. Eight WT kernels (sequence can be cleaved by *Sac* I) and ten *gwt1* kernels (sequence cannot be cleaved by *Sac* I) from segregating ear of *ZmGWT1* heterozygote (+/-) were randomly selected and assayed by dCAPS marker for the single nucleotide mutation from T to C. Scale bar, 1 cm.

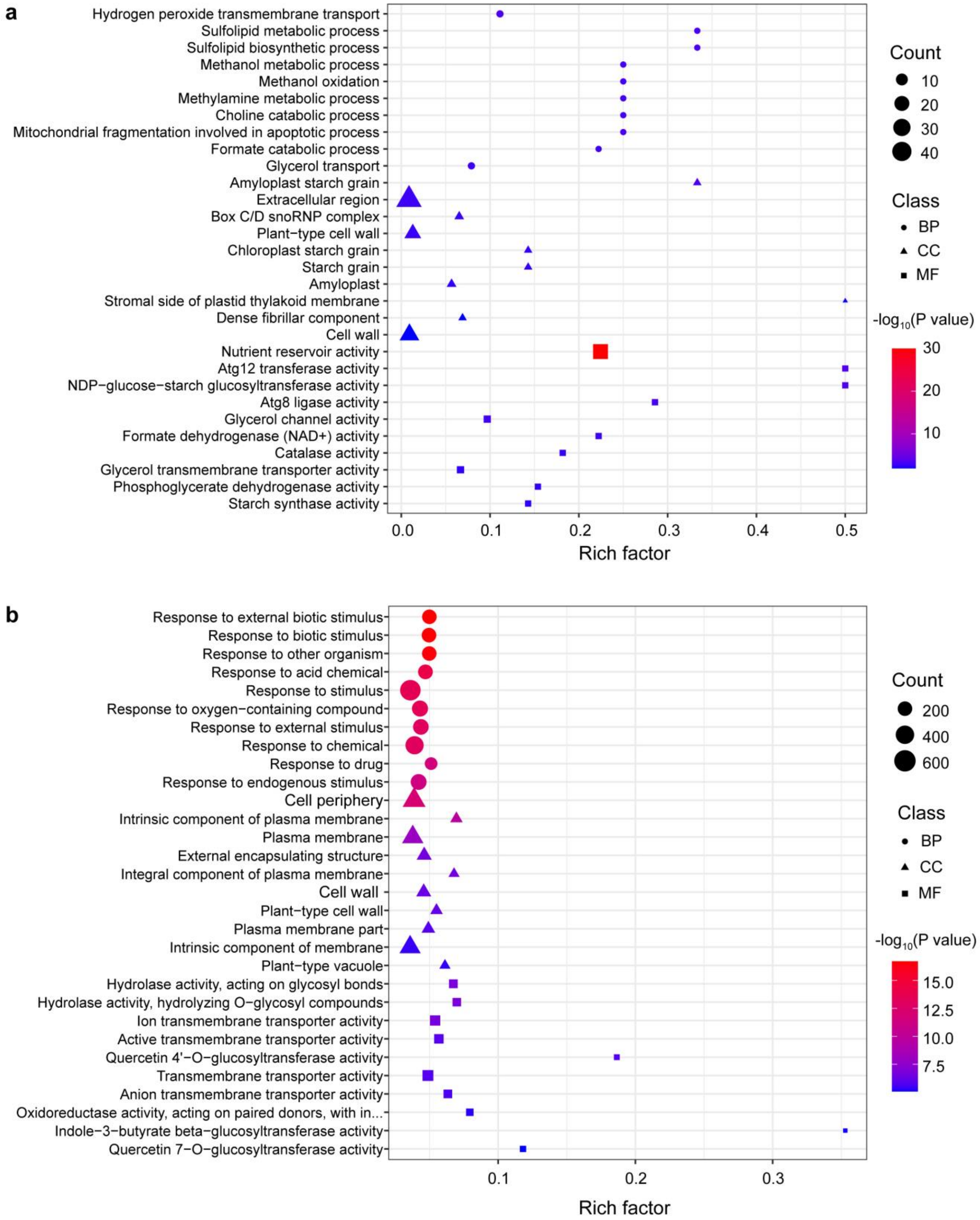

Fig. S2 GO enrichment analysis of DEGs. **a** GO enrichment of down-regulated DEGs in gwt1 compared with WT. **b** GO enrichment of up-regulated DEGs in gwt1 compared with WT.

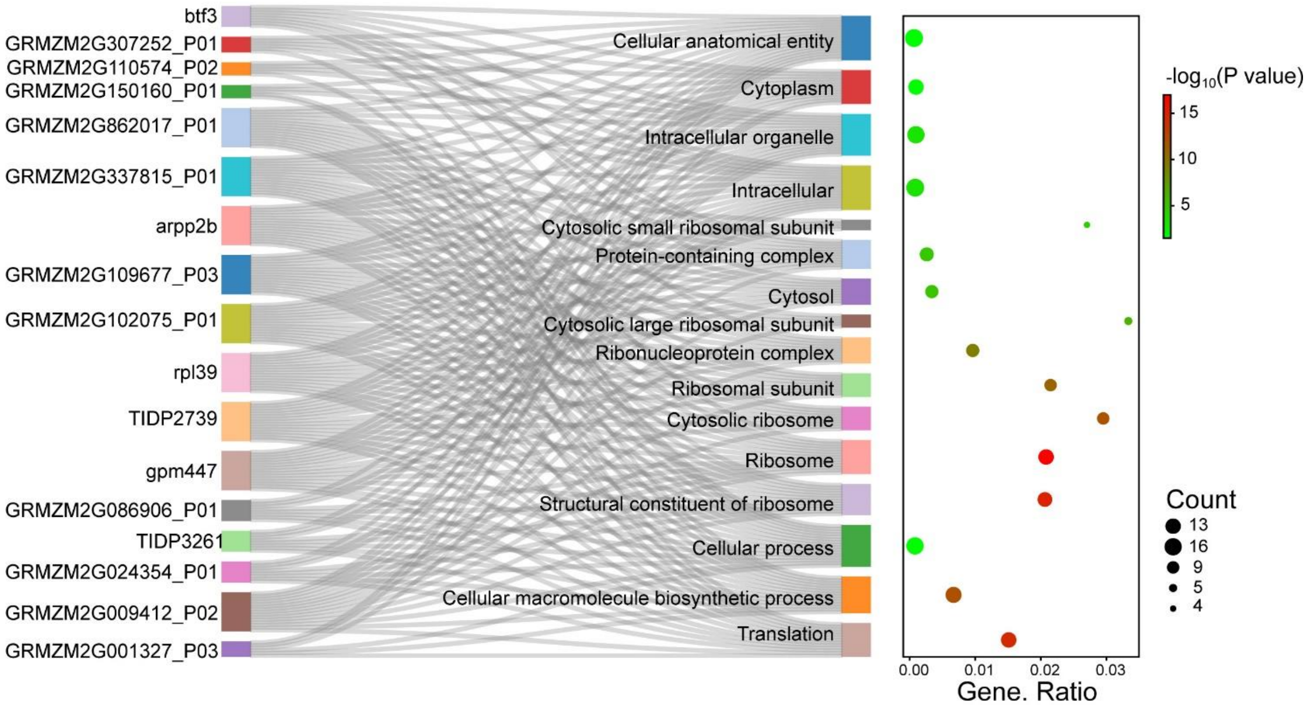

Fig. S3 GO enrichment for PPI network module 1.

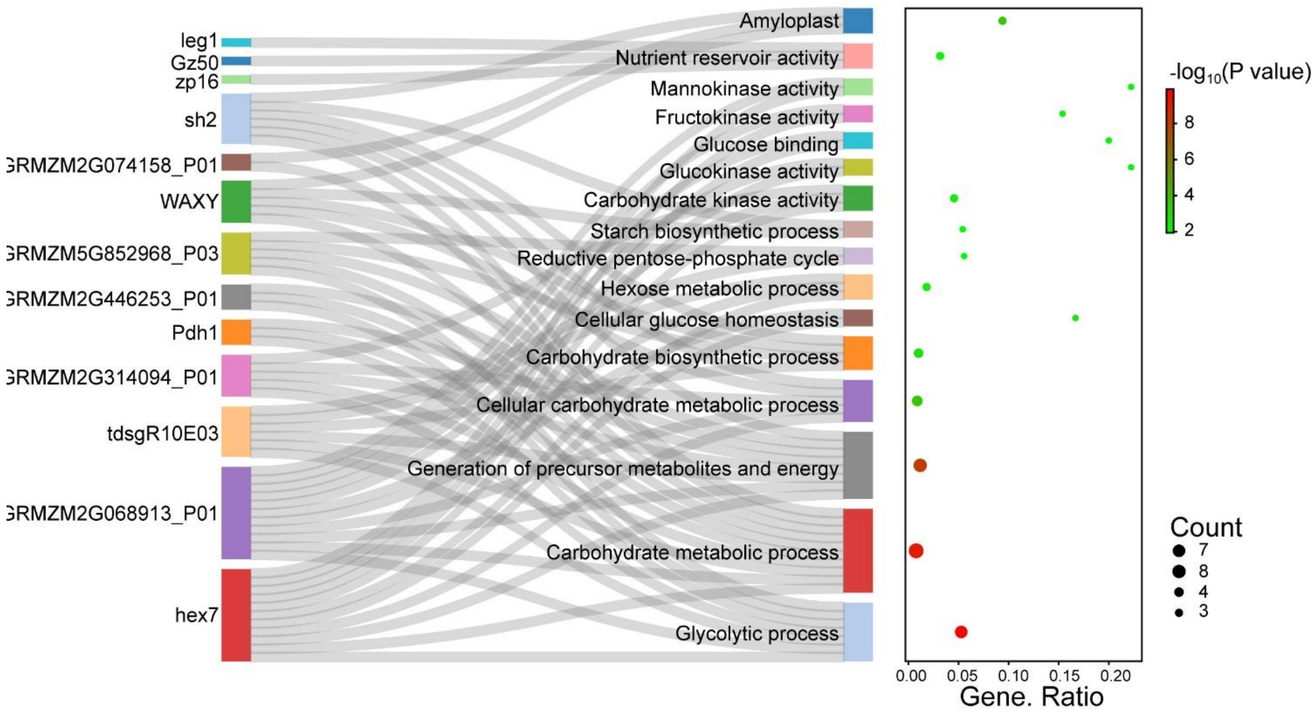

Fig. S4 GO enrichment for PPI network module 2.

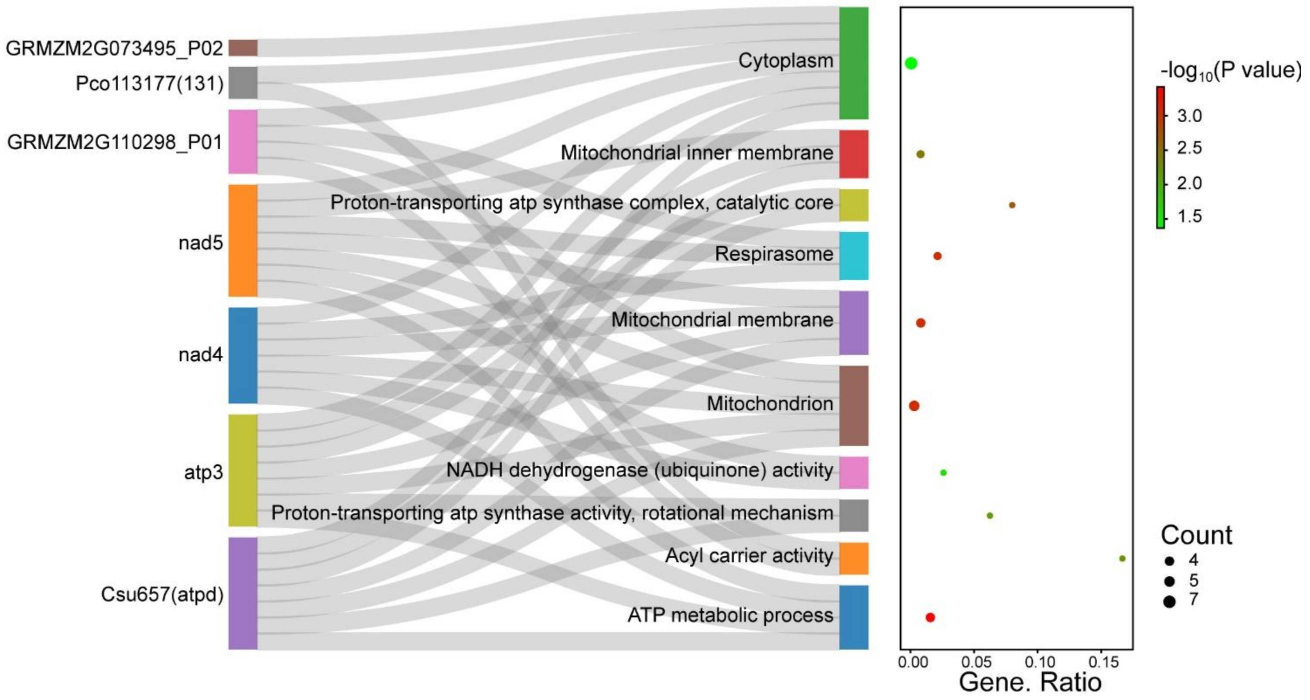

Fig. S5 GO enrichment for PPI network module 3.

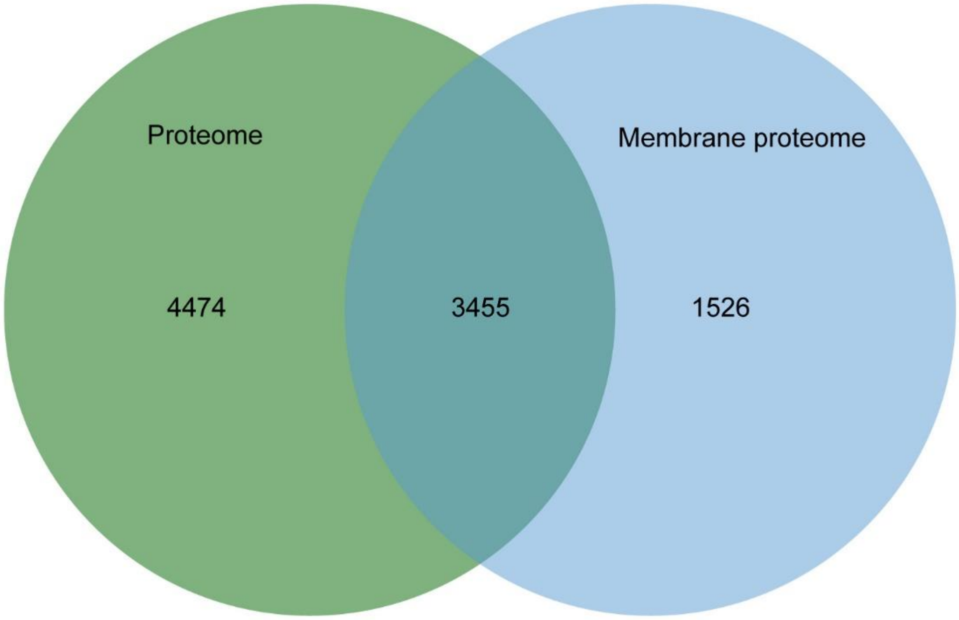

Fig. S6 Integrative analysis of proteome and membrane proteome.

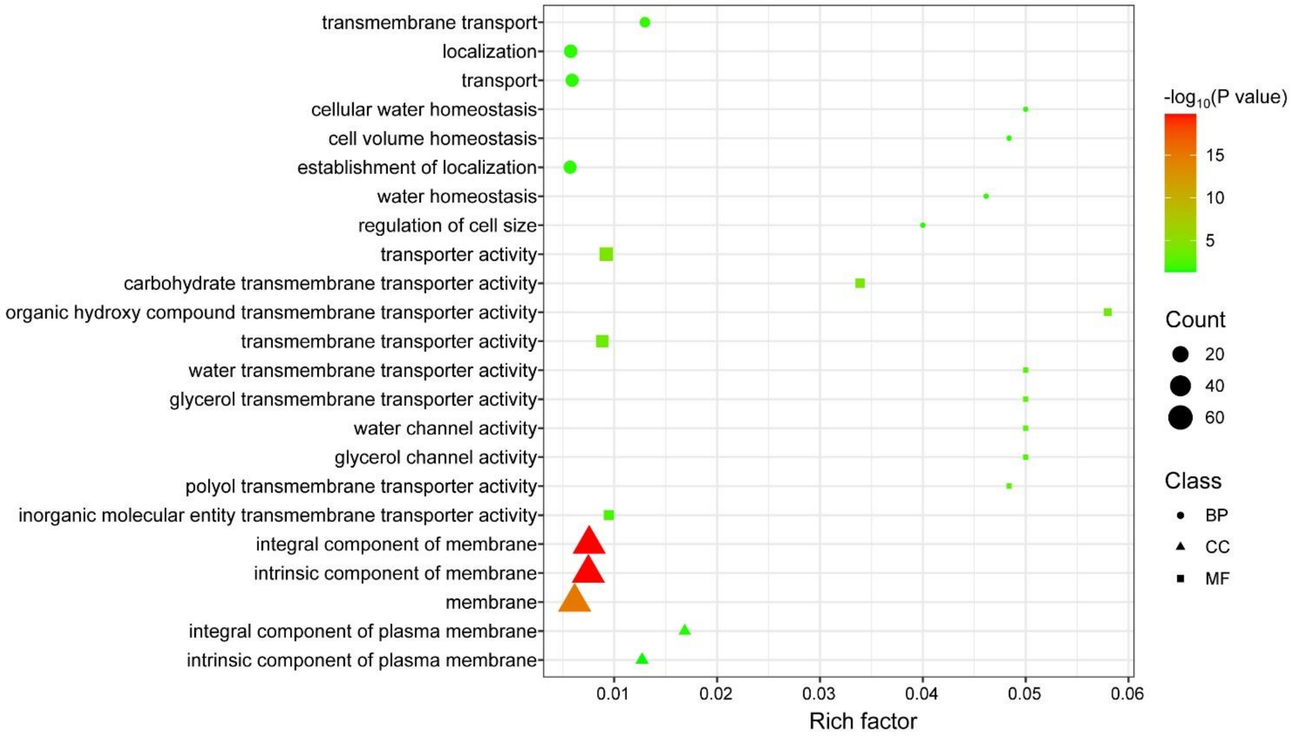

Fig. S7 GO enrichment of differentially accumulated proteins only identified in membrane proteome compared with total proteome.

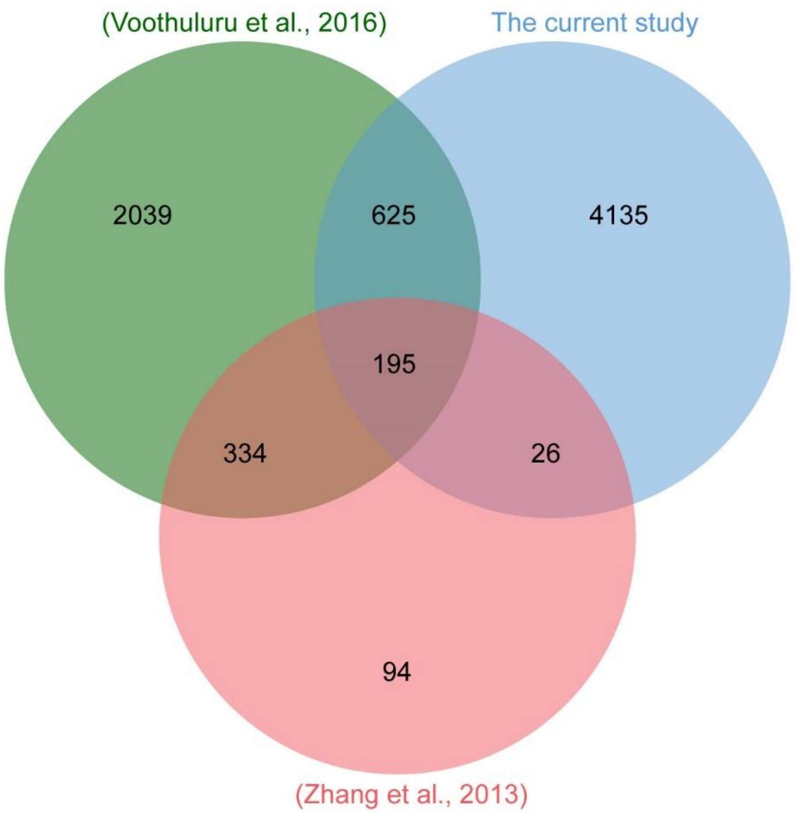

Fig. S8 Comparative analysis of our membrane proteome with previous data.
